# A common regulatory variant in *SLC35B4* influences the recurrence and survival of prostate cancer

**DOI:** 10.1111/jcmm.13649

**Published:** 2018-04-23

**Authors:** Eric Y. Huang, Yu‐Jia Chang, Shu‐Pin Huang, Victor C. Lin, Chia‐Cheng Yu, Chao‐Yuan Huang, Hsin‐Ling Yin, Ta‐Yuan Chang, Te‐Ling Lu, Bo‐Ying Bao

**Affiliations:** ^1^ Department of Urology Taipei Veterans General Hospital Taipei Taiwan; ^2^ Department of Urology School of Medicine National Yang‐Ming University Taipei Taiwan; ^3^ Graduate Institute of Clinical Medicine College of Medicine Taipei Medical University Taipei Taiwan; ^4^ Department of Surgery School of Medicine College of Medicine Taipei Medical University Taipei Taiwan; ^5^ Division of General Surgery Department of Surgery Taipei Medical University Hospital Taipei Medical University Taipei Taiwan; ^6^ Cancer Research Center Taipei Medical University Hospital Taipei Medical University Taipei Taiwan; ^7^ Department of Urology Kaohsiung Medical University Hospital Kaohsiung Taiwan; ^8^ Department of Urology Faculty of Medicine College of Medicine Kaohsiung Medical University Kaohsiung Taiwan; ^9^ Graduate Institute of Medicine College of Medicine Kaohsiung Medical University Kaohsiung Taiwan; ^10^ Institute of Biomedical Sciences National Sun Yat‐sen University Kaohsiung Taiwan; ^11^ Department of Urology E‐Da Hospital Kaohsiung Taiwan; ^12^ School of Medicine for International Students I‐Shou University Kaohsiung Taiwan; ^13^ Division of Urology Department of Surgery Kaohsiung Veterans General Hospital Kaohsiung Taiwan; ^14^ Department of Pharmacy Tajen University Pingtung Taiwan; ^15^ Department of Urology National Taiwan University Hospital College of Medicine National Taiwan University Taipei Taiwan; ^16^ Department of Urology National Taiwan University Hospital Hsin‐Chu Branch Hsinchu Taiwan; ^17^ Department of Pathology Kaohsiung Medical University Hospital Kaohsiung Taiwan; ^18^ Department of Pathology Faculty of Medicine College of Medicine Kaohsiung Medical University Kaohsiung Taiwan; ^19^ Department of Occupational Safety and Health China Medical University Taichung Taiwan; ^20^ Department of Pharmacy China Medical University Taichung Taiwan; ^21^ Sex Hormone Research Center China Medical University Hospital Taichung Taiwan; ^22^ Department of Nursing Asia University Taichung Taiwan

**Keywords:** multi‐stage association study, prognosis, prostate cancer, regulatory variant, SLC35B4

## Abstract

Single nucleotide polymorphisms (SNPs) within the regulatory elements of a gene can alter gene expression, making these SNPs of prime importance for candidate gene association studies. We aimed to determine whether such regulatory variants are associated with clinical outcomes in three cohorts of patients with prostate cancer. We used RegulomeDB to identify potential regulatory variants based on in silico predictions and reviewed genome‐wide experimental findings. Overall, 131 putative regulatory SNPs with the highest confidence score on predicted functionality were investigated in two independent localized prostate cancer cohorts totalling 458 patients who underwent radical prostatectomy. The statistically significant SNPs identified in these two cohorts were then tested in an additional cohort of 504 patients with advanced prostate cancer. We identified one regulatory SNPs, rs1646724, that are consistently associated with increased risk of recurrence in localized disease (*P *=* *.003) and mortality in patients with advanced prostate cancer (*P *=* *.032) after adjusting for known clinicopathological factors. Further investigation revealed that rs1646724 may affect expression of *SLC35B4*, which encodes a glycosyltransferase, and that down‐regulation of *SLC35B4* by transfecting short hairpin RNA in DU145 human prostate cancer cell suppressed proliferation, migration and invasion. Furthermore, we found increased *SLC35B4* expression correlated with more aggressive forms of prostate cancer and poor patient prognosis. Our study provides robust evidence that regulatory genetic variants can affect clinical outcomes.

## 1 INTRODUCTION

Prostate cancer is one of the most common cancers worldwide, and its incidence is increasing in most countries. Despite widespread prostate cancer screening with prostate‐specific antigen (PSA), 15% of patients are still diagnosed at an advanced stage of the disease.^1^ Radical prostatectomy (RP) and androgen deprivation therapy (ADT) are the recommended primary management strategies for patients with localized and advanced prostate cancer, respectively. These therapies are effective in the early stage of the disease, but recurrence and resistance to therapy are the principal causes of morbidity and mortality from prostate cancer. Currently, the defined risk classification of prostate cancer consists of clinical features including PSA level, Gleason score and tumour stage. Although classification is straightforward and useful, patient outcome varies widely. Therefore, additional biomarkers are needed for improved prognosis or early detection to better guide patient management.

**Figure 1:**
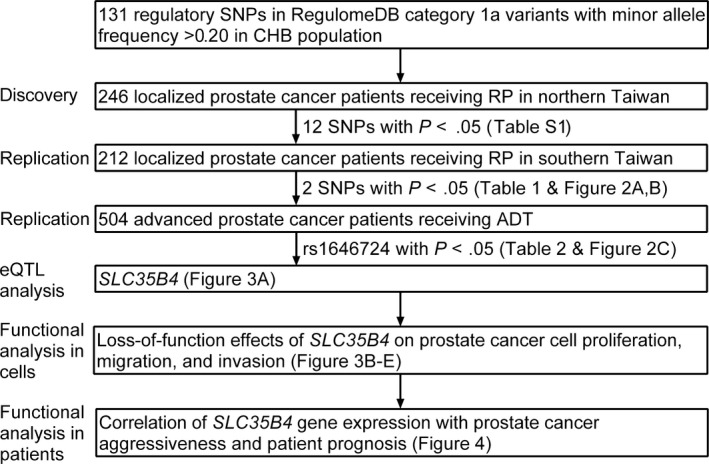
Study design and key findings. The discovery stage 1, replication stage 2 and stage 3, and subsequent functional analyses are illustrated. One candidate susceptibility genes, *SLC35B4*, for prostate cancer progression were identified after replication testing and functional analyses

Considerable progress towards understanding the genetic basis of prostate cancer has been made in recent years because of the development of high‐throughput genotyping technologies.^2^, ^3^ However, most single nucleotide polymorphisms (SNPs) tested in genome‐wide association studies are located outside of the coding regions of genes and therefore have been difficult to interpret.^4^ To identify variants that do not directly affect exonic sequence, but may confer a regulatory effect on gene expression, many studies have mapped open chromatin and protein binding regions for a large number of factors across many cell types.^5^ In addition, computational processing and machine learning have furthered our predictive capabilities in identifying regulatory variants. We hypothesized that such functional regulatory SNPs have biologically important functions that may have an effect on cancer progression. In this multi‐stage study, we systematically evaluated a comprehensive panel of predicted and known regulatory SNPs to determine their roles in prostate cancer progression and patient mortality, as illustrated in Figure 1.

## 2 MATERIALS AND METHODS

### 2.1 Patient recruitment and data collection

This study included 458 patients with localized prostate cancer who underwent RP as initial therapy and 504 patients with advanced prostate cancer who received primary ADT, as described previously.^6^, ^7^, ^8^, ^9^, ^10^, ^11^ The localized prostate cancer cohort consisted of participants from two independent data sets. The discovery data set was composed of 246 patients from the National Taiwan University Hospital located in northern Taiwan, and the replication data set was composed of 212 patients from the Kaohsiung Medical University Hospital, the E‐Da Hospital and the Kaohsiung Veterans General Hospital, all located in southern Taiwan. Clinical and follow‐up data were obtained from patient medical records. The primary end‐point was biochemical recurrence (BCR), which was defined as two consecutive PSA values of at least 0.2 ng/mL after RP.^12^, ^13^ Patients in the advanced prostate cancer cohort were recruited from all four medical centres. Overall survival (OS) was defined as the duration from diagnosis to death of any cause or to last follow‐up. This study was approved by the Institutional Review Boards of the National Taiwan University Hospital, the Kaohsiung Medical University Hospital, the E‐Da Hospital and the Kaohsiung Veterans General Hospital. Written informed consent was obtained from each patient prior to their participation in the study, which was performed in accordance with the approved guidelines.

### 2.2 SNP selection and genotyping

The RegulomeDB database (http://www.regulomedb.org) uses a scoring system to guide interpretation of putatively regulatory variants based on their functional confidence.^14^ Category 1a variants, which are classified as most likely to affect binding and likely to alter expression of a target gene, have the following characteristics: (i) known expression quantitative trait loci (eQTL) for genes, (ii) change transcription factor binding, (iii) are found in a transcription factor motif, (iv) and have a DNase footprint. We screened common (minor allele frequency > 0.2) regulatory SNPs by comparing 352 RegulomeDB category 1a variants with the HapMap Han Chinese in Beijing, China (CHB) population,^15^ and identified 164 common regulatory class 1a SNPs.

Genomic DNA was extracted from peripheral blood samples from participants using the QIAamp DNA Blood Mini Kit (Qiagen, Valencia, CA) and stored at −80°C. Genotyping was performed at the National Center for Genome Medicine (Taiwan) using the Agena Bioscience MassARRAY iPLEX system (Agena Bioscience, San Diego, CA), as described previously.^9^ The average genotype call rate for these SNPs was 98.9%, and the concordance rate was 100% among 10 blind duplicated quality control samples. Any SNP that failed the assay design (N* *=* *18), deviated from Hardy‐Weinberg equilibrium (*P *<* *.005, N* *=* *13), or had a genotyping call rate <85% (N* *=* *2), was removed. Therefore, 131 SNPs underwent statistical analysis.

### 2.3 Tissue samples and immunohistochemistry

Two tissue microarrays of prostate adenocarcinoma were purchased from SuperBioChips Laboratories (catalogue no.: CA4, Seoul, South Korea) and US Biomax (catalogue no.: PR8011a, Rockville, MD). Seven normal, thirteen adjacent normal, six inflammation, twenty‐six hyperplasia and seventy‐one adenocarcinoma tissues were available for the immunohistochemical study of SLC35B4 with these two tissue microarrays. Sections were deparaffinized, rehydrated and blocked with 3% hydrogen peroxide. Heat‐induced antigen retrieval was performed in citric acid buffer (pH 6.0) at 121°C for 10 minutes using a decloaking chamber (Biocare Medical, Concord, CA). Next, the sections were incubated with a rabbit polyclonal SLC35B4 antibody (1:200, catalogue no.: NBP2‐13329, Novus Biologicals, Littleton, CO) at 4°C overnight. SLC35B4 expression was detected using a Starr Trek Universal HRP Detection System (Biocare Medical). The antigen was identified with addition of 3′‐diaminobenzidine, followed by haematoxylin counterstaining. Appropriate positive and negative controls were included in these assays. The intensity of SLC35B4 expression in tumour cells was scored semi‐quantitatively as weak expression (1+), moderate expression (2+) or strong expression (3+).

### 2.4 Bioinformatics analysis

We used several bioinformatics tools to assess whether rs1646724 or linked genetic variants were associated with a putative function that may have an effect on patient outcome. Tests of association between rs1646724 and *SLC35B4* expression were evaluated using mRNA data of lymphoblastoid cell lines derived from HapMap and consisting of 90 Utah residents with ancestry from northern and western Europe (CEU), 45 CHB, 45 Japanese in Tokyo, Japan (JPT) and 90 Yoruba in Ibadan, Nigeria (YRI).^16^ HaploReg v4.1^17^ data were used to evaluate the regulatory potential of the region flanking the SNPs tested. The publicly available MSKCC Prostate Oncogenome Project data sets^18^ were used to analyse *SLC35B4* expression and patient prognosis.

### 2.5 Chemicals, regents, generation of *SLC35B4* knockdown prostate cancer cells, evaluation of cancer cell proliferation, migration and invasion and Western blot analysis

PC‐3 (CRL‐1435) and DU145 (HTB‐81) human prostate cancer cell lines were purchased from ATCC (Manassas, VA) and maintained in the recommended culture media. The identity of the cell line was checked by Cell ID System and Promega GenePrint 10 System through short tandem repeat analysis (Mission Biotech, Taipei, Taiwan), and passage numbers used for the experiments are <25. A comprehensive methods section is available in Supplementary methods.

### 2.6 Statistical analysis

Patient clinicopathological characteristics were summarized as either the number and proportion (%) of patients or as the median and interquartile range. Evidence of association between clinicopathological characteristics and either BCR or OS was assessed using the log‐rank test or Cox regression. Multivariate Cox proportional hazards regression analyses were used to assess the effect of each SNP on clinical outcomes with adjustments for clinicopathological variables, as described previously.^13^, ^19^, ^20^, ^21^, ^22^, ^23^, ^24^, ^25^ In the localized prostate cancer cohort, multiple explanatory variables included known prognostic factors: age, PSA at diagnosis, pathologic Gleason score and pathologic stage. In the advanced prostate cancer cohort, multiple explanatory variables included known prognostic factors: age, PSA at ADT initiation, Gleason score, clinical stage, PSA nadir and treatment modality. We compared three genetic models of inheritance to evaluate the significance of each SNP tested: dominant (major allele homozygous genotype versus pooled heterozygous and minor allele homozygous genotypes), recessive (pooled major allele homozygous and heterozygous genotypes versus minor allele homozygous genotype) and additive (*P* for trend). Only dominant and additive modes of inheritance were considered if the frequency of the minor allele homozygous genotype was <0.05 in the study population. Fixed‐effects and random‐effects meta‐analyses were performed to calculate the combined hazard ratios (HRs). Heterogeneity between cohorts was evaluated by Cochran's χ^2^‐based *Q* test. If the results of the *Q* test were significant, a random‐effects model was used to accommodate the diversity; otherwise, the combined HR was estimated using a fixed‐effects model. The Statistical Package for the Social Sciences software v22.0.0 (IBM, Armonk, NY) was used for statistical analyses. A two‐sided *P* value <.05 was considered statistically significant.

## 3 RESULTS

Patient characteristics are shown in Table 1. For the discovery and replication cohorts with localized prostate cancer, 30.5% (75/246) and 51.4% (109/212) of patients experienced BCR at median follow‐ups of 50 and 60 months, respectively. We found that BCR was associated with PSA, pathological Gleason score and tumour stage (all *P *<* *.001). For the advanced prostate cancer cohort, patient mortality was 29.4% (148/504) at a median follow‐up of 64 months. In addition, we found that OS was associated with PSA at ADT initiation, Gleason score, clinical stage at diagnosis, PSA nadir and treatment modality (all *P *≤* *.001).

**Table 1:** Clinical characteristics of study cohorts

Characteristic	Discovery	Replication	Combined	*P* a
Localized prostate cancer cohort				
Patients, n	246	212	458	
Age at diagnosis				
Median, y (IQR)	65 (61‐69)	68 (62‐71)	66 (61‐70)	.149
PSA at diagnosis				
Median, ng/mL (IQR)	10.1 (6.7‐15.8)	12.6 (7.6‐19.8)	11.1 (7.1‐17.5)	<.001
≤20	192 (81.7)	155 (76.0)	347 (79.0)	
>20	43 (18.3)	49 (24.0)	92 (21.0)	
Pathologic Gleason score, n (%)				
≤7	217 (89.7)	175 (82.9)	392 (86.5)	<.001
>7	25 (10.3)	36 (17.1)	61 (13.5)	
Pathologic stage, n (%)				
T1/T2	173 (72.4)	130 (61.3)	303 (67.2)	<.001
T3/T4/N1	66 (27.6)	82 (38.7)	148 (32.8)	
BCR	75 (30.5)	109 (51.4)	184 (40.2)	
Median follow‐up time^b^, mo (95% CI)	50 (45‐55)	60 (56‐64)	54 (50‐58)	

Characteristic		*P* c
Advanced prostate cancer cohort		
Patients, n	504	
Age at diagnosis		
Median, y (IQR)	73 (66‐79)	.078
PSA at ADT initiation		
Median, ng/mL (IQR)	33.8 (9.3‐133.3)	<.001
<35	253 (51.6)	
≥35	237 (48.4)	
Biopsy Gleason score at diagnosis, n (%)		
≤7	312 (63.4)	<.001
>7	180 (36.6)	
Clinical stage at diagnosis, n (%)		
M0	308 (61.4)	<.001
M1	194 (38.6)	
PSA nadir		
Median, ng/mL (IQR)	0.14 (0.01‐1.06)	<.001
Treatment modality		
ADT as primary treatment	254 (50.5)	.001
ADT for post‐RP PSA failure	73 (14.5)	
ADT for post‐RT PSA failure	12 (2.4)	
Neoadjuvant/adjuvant ADT with RT	122 (24.3)	
Others	42 (8.3)	
Deceased	148 (29.4)	
Median follow‐up time^b^, mo (95% CI)	64 (60‐68)	

IQR, interquartile range; PSA, prostate‐specific antigen; BCR, biochemical recurrence; CI, confidence interval; ADT, androgen deprivation therapy; RP, radical prostatectomy; RT, radiation therapy.

a *P* value was calculated by the log‐rank test or Cox regression for BCR in combined 458 localized prostate cancer patients.

b Median follow‐up time and 95% CIs were estimated with the reverse Kaplan‐Meier method.

c *P* value was calculated by the log‐rank test or Cox regression for overall survival in advanced prostate cancer patients.

**Figure 2:**
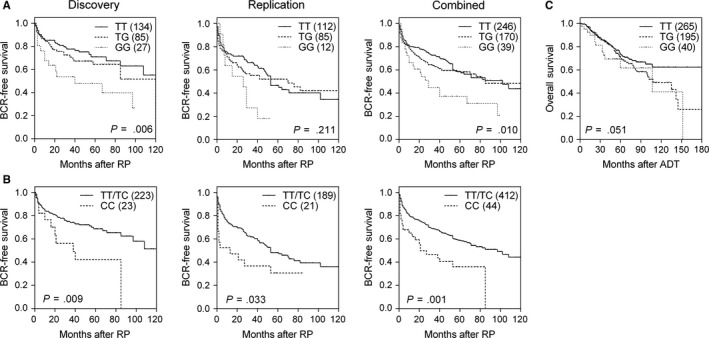
Impact of rs1646724 and rs4239504 on prostate cancer prognosis. Kaplan‐Meier estimates of BCR‐free survival by (A) rs1646724 and (B) rs4239504 genotype for patients with localized prostate cancer receiving RP in the discovery cohort, replication cohort and combined analysis. (C) Kaplan‐Meier estimates of overall survival by rs1646724 genotype for patients with advanced prostate cancer receiving ADT. Numbers in parentheses indicate the number of patients

Of the 131 regulatory SNPs that were genotyped, we found 12 SNPs were associated with BCR in the discovery data set of patients with localized prostate cancer (all *P *<* *.05, Table S1). This subset of significant SNPs was tested using the replication data set, and two SNPs, rs1646724 and rs4239504, were validated in the replication cohort (*P *≤* *.047) and in the combined analysis (*P *≤* *.005; Table 2). After adjusting for age, PSA, pathological Gleason score and stage, we found that rs1646724 and rs4239504 remained significant (*P *≤* *.009). The minor G allele of rs1646724 was associated with an increased risk of BCR in a dose‐dependent manner [HR 1.38, 95% confidence interval (CI) 1.09‐1.74, *P *=* *.007; Table 2 and Figure 2A]. In addition, patients with the rs4239504 CC genotype had a 1.78‐fold increased risk of BCR (95% CI 1.15‐2.75, *P *=* *.009; Table 2 and Figure 2B). Concordant with our findings in patients with localized prostate cancer, we found that rs1646724 was also significantly associated with OS in patients with advanced prostate cancer after adjusting for age, PSA at ADT initiation, Gleason score, stage, PSA nadir and treatment modality (HR 1.35, 95% CI 1.03‐1.78, *P *=* *.032; Table 3 and Figure 2C).

**Table 2:** Association of regulatory genetic variants with BCR in patients with localized prostate cancer treated with RP

SNP Genotype	Discovery				Replication				Combined			
	n	BCR	HR (95% CI)	*P*	n	BCR	HR (95% CI)	*P*	HR (95% CI)	*P*	HR (95% CI)^a^	*P* a
rs1646724												
TT	134	34	1.00		112	56	1.00		1.00		1.00	
TG	85	27	1.35 (0.81‐2.25)	.245	85	41	1.03 (0.69‐1.54)	.905	1.14 (0.83‐1.56)	.41	1.20 (0.86‐1.67)	.28
GG	27	14	**2.52 (1.35‐4.72)**	**.004**	12	9	**2.04 (1.00‐4.14)**	**.050**	**2.30 (1.44‐3.68)**	**.0005**	**2.23 (1.36‐3.67)**	**.002**
TG/GG vs TT			**1.61 (1.02‐2.54)**	**.042**			1.13 (0.77‐1.65)	.544	1.31 (0.98‐1.75)	.07	1.35 (0.99‐1.83)	.06
GG vs TT/TG			**2.23 (1.24‐4.00)**	**.007**			**2.02 (1.01‐4.02)**	**.047**	**2.14 (1.37‐3.35)**	**.0009**	**2.03 (1.27‐3.25)**	**.003**
Trend			**1.54 (1.13‐2.11)**	**.007**			1.22 (0.89‐1.67)	.218	**1.37 (1.10‐1.71)**	**.005**	**1.38 (1.09‐1.74)**	**.007**
rs4239504												
TT	121	43	1.00		91	44	1.00		1.00		1.00	
TC	102	21	0.60 (0.35‐1.01)	.054	98	49	0.92 (0.61‐1.38)	.688	0.79 (0.57‐1.09)	.15	0.86 (0.61‐1.20)	.38
CC	23	11	1.86 (0.96‐3.63)	.068	21	14	1.74 (0.95‐3.17)	.073	**1.79 (1.15‐2.80)**	**.01**	**1.66 (1.05‐2.62)**	**.03**
TC/CC vs TT			0.78 (0.49‐1.23)	.284			1.03 (0.70‐1.51)	.890	0.92 (0.68‐1.24)	.58	0.99 (0.73‐1.35)	.97
CC vs TT/TC			**2.29 (1.20‐4.36)**	**.012**			**1.82 (1.03‐3.19)**	**.038**	**2.01 (1.31‐3.08)**	**.001**	**1.78 (1.15‐2.75)**	**.009**
Trend			1.04 (0.72‐1.49)	.839			1.17 (0.87‐1.59)	.308	1.12 (0.89‐1.41)	.35	1.14 (0.90‐1.44)	.27

BCR, biochemical recurrence; RP, radical prostatectomy; SNP, single nucleotide polymorphism; HR, hazard ratio; CI, confidence interval; PSA, prostate‐specific antigen.

a Adjusted by age, PSA at diagnosis, pathologic Gleason score and pathologic stage.

*P *<* *.05 are in boldface.

**Table 3:** Association of rs1646724 with ACM in patients with advanced prostate cancer treated with ADT

SNP Genotype	n	ACM	5‐year survival rate, %	HR (95% CI)^a^	*P* a
rs1646724					
TT	265	69	73.0	1.00	
TG	195	64	70.5	1.27 (0.89‐1.81)	.193
GG	40	14	61.6	**2.03 (1.07‐3.83)**	**.030**
TG/GG vs TT				1.35 (0.95‐1.90)	.091
GG vs TT/TG				1.80 (0.98‐3.30)	.058
Trend				**1.35 (1.03‐1.78)**	**.032**

ACM, all‐cause mortality; ADT, androgen deprivation therapy; HR, hazard ratio; CI, confidence interval; PSA, prostate‐specific antigen.

a Adjusted by age, PSA at ADT initiation, Gleason score, stage, PSA nadir and treatment modality.

*P *<* *.05 are in boldface.

**Figure 3:**
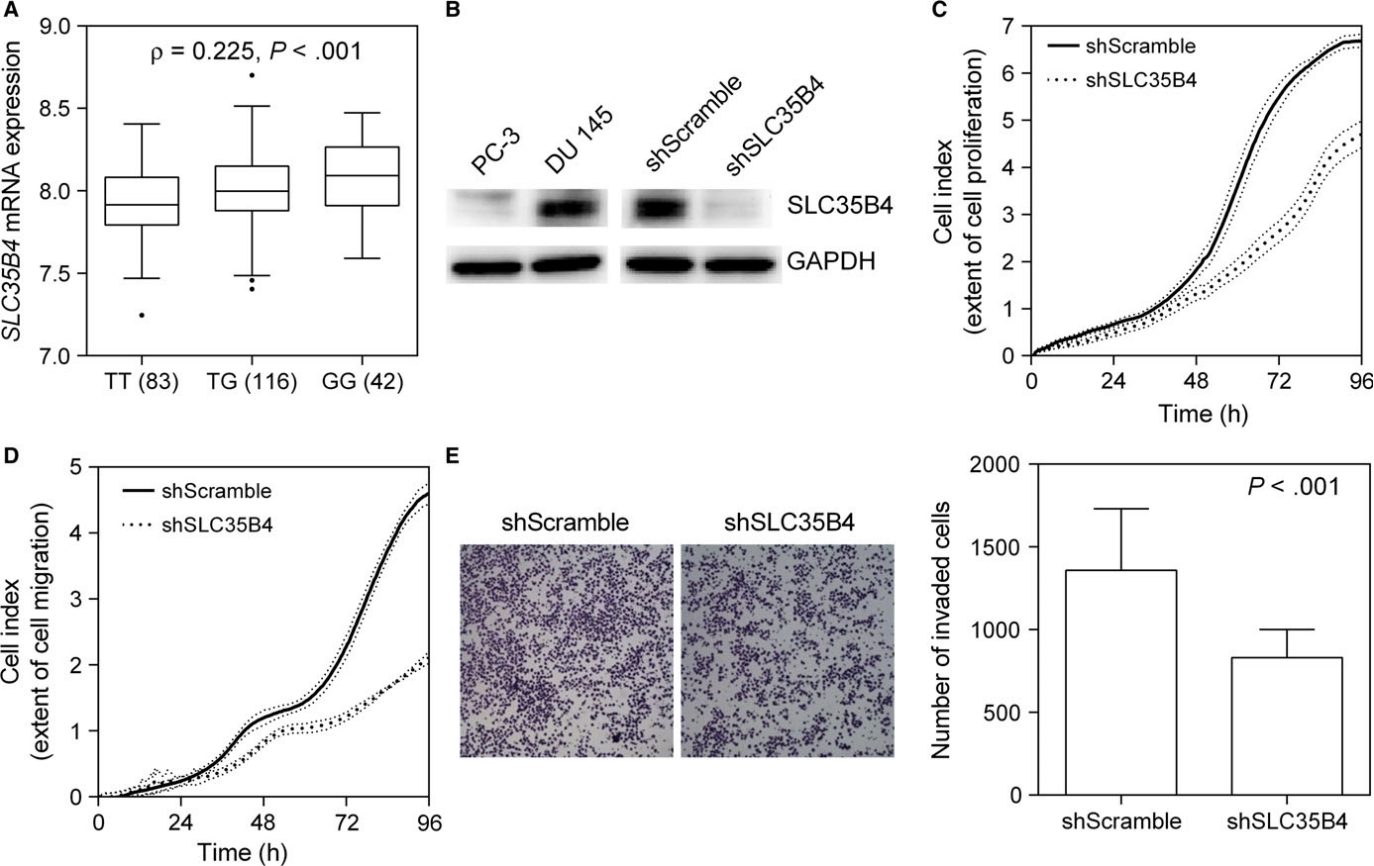
Silencing *SLC35B4* expression inhibits DU145 cell growth and migration. (A) Correlation of rs1646724 genotype with *SLC35B4* expression. There is a trend towards increased SLC35B4 mRNA expression in carriers of the rs1646724 risk allele G. The numbers in parentheses indicate the number of cases. (B) Western blots showing that expression of SLC35B4 in DU145 cells is increased compared to that found in PC‐3 cells, and that expression was effectively down‐regulated by transfecting shRNA into DU145 cells. Down‐regulation of *SLC35B4* inhibits DU145 cell (C) proliferation, (D) migration and (E) invasion. Data are expressed as the mean ± standard deviation (SD) of three independent experiments

The regulatory variant rs1646724 is located 71 base pair (bp) upstream to the *SLC35B4* start site. Thus, we performed an in silico analysis using genetic variation and gene expression data from HapMap populations to determine whether rs1646724 could affect *SLC35B4* expression. From our analysis, a positive Spearman correlation coefficient indicated that the rs1646724 risk allele G is correlated with increased *SLC35B4* expression (*P *<* *.001, Figure 3A). As an initial step towards understanding the role of SLC35B4 in prostate cancer, we evaluated whether SLC35B4 has a role in proliferation, migration and invasion of prostate cancer cells. First, using Western blot analysis, we examined the protein level of SLC35B4 in cells from two widely used metastatic human prostate cancer cell lines, PC‐3 and DU145. As shown in Figure 3B, we found increased SLC35B4 protein in DU145 cells compared to that found in PC‐3 cells. Next, using a short hairpin RNA (shRNA) approach, we performed *SLC35B4* knockdown in DU145 cells and found that the protein level of SLC35B4 was effectively down‐regulated in *SLC35B4* knockdown cells (shSLC35B4) compared to cells with a scrambled control (shScramble; Figure 3B). We then evaluated cell proliferation and migration using a real‐time xCELLigence biosensor system for 96 hours. We found that SLC35B4 depletion significantly reduced DU145 cell proliferation (Figure 3C) and migration (Figure 3D). In addition, we used an invasion assay to measure the capability of cancer cells to degrade and invade a basement membrane matrix. We found that the number of invading cells was dramatically decreased in shSLC35B4 cells compared to shScramble control cells (Figure 3E). These findings demonstrate that SLC35B4 may be important in prostate cancer progression.

**Figure 4:**
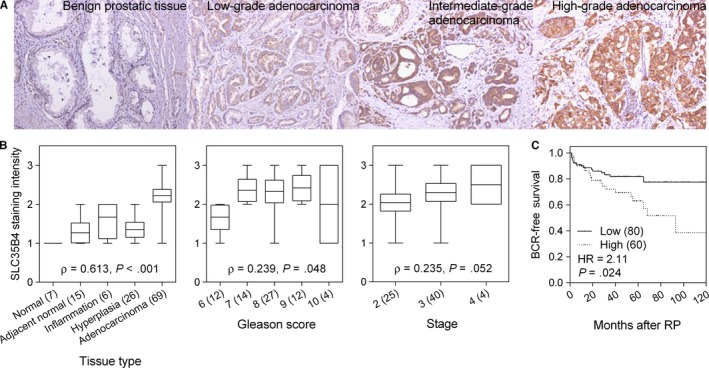
Increased SLC35B4 expression is correlated with prostate cancer aggressiveness and poor patient prognosis. (A) Representative images of immunohistochemical staining for SLC35B4 expression in human benign prostate tissues as well as low‐, intermediate‐, and high‐grade adenocarcinomas. (B) Correlation of SLC35B4 expression with prostate cancer progression. More advanced prostate cancers and a higher Gleason score display a tendency towards increased SLC35B4 expression. (C) Increased *SLC35B4* mRNA expression is correlated with reduced BCR‐free survival in patients with prostate cancer. Numbers in parentheses indicate the number of patients

To understand the clinical relevance of SLC35B4 expression in prostate cancer, we examined SLC35B4 expression in a large number of prostate carcinoma cases using tissue microarrays. Representative images of SLC35B4 immunostaining are shown in Figure 4A. We found a trend towards increased SLC35B4 expression with more aggressive forms of prostate cancer (*P *<* *.001, Figure 4B), and that up‐regulation of SLC35B4 was correlated with higher Gleason tumour scores (*P *=* *.048, Figure 4B). Moreover, our analysis of an independent public data set showed that high *SLC35B4* mRNA levels also signified poor BCR‐free survival (Figure 4C). Our results suggest that SLC35B4 warrants further investigation as a potential prognostic marker of prostate cancer.

## 4 DISCUSSION

We systematically analysed a large panel of SNPs residing in regulatory regions that could modulate transcription factor binding, and subsequently, affect target gene expression. In our investigation using three different prostate cancer cohorts, we identified one SNP, rs1646724, in *SLC35B4* that was consistently associated with recurrence in patients with localized disease and OS in patients with advanced prostate cancer. Using a combination of different in vivo and in vitro assays, we demonstrated that *SLC35B4* expression was increased in prostate cancer tissues and that silencing *SLC35B4* expression suppressed cancer cell proliferation, migration and invasion. Our experimental findings were further supported by our analysis of publicly available datasets, which showed that a high level of SLC35B4 mRNA is linked to poor prognosis in patients with prostate cancer.

Based on HaploReg data,^26^ rs1646724 is located 71 bp upstream of the *SLC35B4* start site and is predicted to lie within a transcription regulatory region containing putative binding sites for BCL2 associated transcription factor 1 (BCLAF1) and zinc finger protein 143 (ZNF143). In both cases, the rs1646724 risk allele G creates the binding site motif for BCLAF1 and ZNF143, and therefore, may induce or increase *SLC35B4* expression (Figure 3A). *SLC35B4*, known as solute carrier family 35 member B4, encodes a glycosyltransferase that transports UDP‐xylose and UDP‐N‐acetylglucosamine from the cytosol to the Golgi apparatus where they are utilized in the synthesis of glycoproteins, glycolipids and proteoglycans.^27^ Although *SLC35B4* has not been linked to cancer, alterations in the glycosylation patterns of cancers were confirmed following the development of antibodies against tumour glycoproteins.^28^, ^29^ The expression of cancer‐associated glycans, such as sialyl‐Lewis^X^, Thomsen‐nouvelle (Tn) antigen and sialyl‐Tn antigen, has been detected in many types of cancer, including prostate cancer.^30^ Glycosylation is a frequent post‐translational protein modification, and the degree of protein glycosylation depends on the glycosylation sites and the expression and activity of glycosyltransferases such as SLC35B4, and glycosidase enzymes. It is established that glycomodification can modulate activity of numerous growth factor receptors including EGFR, PDGFR and IGFR, all of which can sustain proliferative signaling.^31^ Cancer cells often have high levels of sialylated glycans,^32^ and increased sialylation can increase local negative charges to disrupt cell–cell adhesion.^33^ Expression of the cancer‐associated sialyl‐Tn antigen reduces cell adhesion and increases migration and invasion in prostate cancer.^34^ Because the prostate is a secretory gland involved in the secretion of many types of glycoproteins, alterations in glycans are of particular interest as potential biomarkers and therapeutic targets for prostate cancer. Consistent with these observations, we found that increased *SLC35B4* expression was associated with more aggressive forms of prostate cancer (Figure 4), and that silencing *SLC35B4* markedly suppresses cancer cell proliferation, migration and invasion (Figure 3). Based on these findings, we posit that SLC35B4 may alter glycosylation thereby modifying some biological processes involved in prostate cancer progression.

The main limitations of this study were that the sample size of our separate cohorts was modest and that multiple statistical comparisons were made. Even with independent discovery and replication cohorts study design to validate our findings and reduce the possibility of a false discovery, our results should still be interpreted with caution owing to the number of statistical tests performed. In addition, using functional assays provided confirmatory support of gene‐disease associations. Another limitation of our study was that all participants were Taiwanese, and because of homogeneity of the Taiwanese population, our findings may be less generalizable to other ethnic groups. However, the same homogeneity also enables the identification of unique ethnic‐ or population‐specific culprit genes that may not be found in association studies of other groups. Additional research is warranted to investigate the biological mechanisms that underlie our observed associations.

In summary, this study provides evidence for an association between the regulatory variant *SLC35B4* rs1646724 and the clinical outcomes of patients with prostate cancer, which may be of prognostic or therapeutic significance. Future studies are warranted to validate our findings in a larger cohort and in other ethnic groups and populations, and to determine the functional importance of SLC35B4 in prostate cancer progression. Nevertheless, if validated, these regulatory variants or related markers in *SLC35B4* may lead to better patient stratification, optimize therapeutic interventions in high‐risk patients and elucidate new cancer drug targets.

## CONFLICT OF INTEREST

The authors declare that they have no conflict of interest.
