# Supplementary material for: A common regulatory variant in SLC35B4 influences the recurrence and survival of prostate cancer
Source: J Cell Mol Med. 2018 Apr 23;22(7):3661–70. doi: 10.1111/jcmm.13649 (PMC6010704; doi:10.1111/jcmm.13649)
Supplement: Supplementary file 1 [file JCMM-22-3661-s001.doc]

**Supplementary methods**

**Chemicals, regents, and cell culture**

Human prostate cancer PC-3 and DU145 cells were maintained in RPMI-1640 (Gibco BRL, Grand Island, NY) with 2 mM L-glutamine, 1.5 g/L sodium bicarbonate, 10% fetal calf serum (FCS, Gibco BRL) and 2% penicillin-streptomycin (10,000 U/mL penicillin and 10 mg/mL streptomycin). Cells were maintained in a 5% CO_2_ humidified incubator at 37 °C as described previously [1,2]. Anti-SLC35B4 antibody (NBP2-13329) was purchased from Novus Biologicals (Littleton, CO) and anti-GAPDH (sc32233) was purchased from Santa Cruz Biotechnology (Santa Cruz, CA).

**Generation of *SLC35B4* knockdown prostate cancer cells**

The plasmid parental vector (pLKO.1-puro) containing *SLC35B4*-specific short hairpin RNA (shRNA) (National RNAi Core Facility, Academia Sinica, Taiwan) was transfected into cancer cells and selected by puromycin. The target sequence for the human SLC35B4 mRNA (NM_032826) was 5′-CGGTTCTCTAATTGCCAACAT-3′. The MISSION non-target shRNA control vector (SHC002) was used as a scrambled control (Sigma Chemicals) [1-4]. The generation of *SLC35B4* knockdown cells was modified from previous publications [3-5]. Briefly, 1.5 × 10^5^ cells were mixed with 0.5 μg of plasmid after washed twice by phosphate-buffered saline (PBS). One pulse for a duration of 20 ms under a fixed voltage of 1.4 kV was applied by a Neon pipette-type microporator (Invitrogen Life Technologies, Grand Island, NY), and the stably transfected cell lines were selected using puromycin (1 mg/mL) as described previously.

**Evaluation of cancer cell proliferation and migration**

Experiments were performed using an xCELLigence RTCA DP instrument (ACEA Biosciences, San Diego, CA), which was placed in an incubator with 5% CO_2_ at 37 °C. Our experiments were modified from a previous study [6]. Cell growth was performed on an E-plate 16 (ACEA Biosciences). We seeded 1 × 10^4^ cells/well on an E-plate 16 in FCS-containing medium. The plate was monitored once every 30 s for 4 h followed by once every 30 min. Cell migration was assessed using specifically designed CIM-plate 16 with 8 μm pores. After adding 10% FCS medium to the lower chamber, cells were seeded into the upper chamber at 2 × 10^4^ cells/well in serum-free medium. The CIM-plate 16 was monitored every 10 s for 40 min and once every hour thereafter. Data were analyzed using RTCA software v1.2.

***In vitro* invasion assay**

Cell motility was assessed using a 24-well BD BioCoat™ Matrigel Invasion Chamber (BD Biosciences). Cells (1 x 10^5^) suspended in 500 μL of serum-free RPMI were seeded into the upper compartments of each chamber, and the lower compartments were filled with 1 mL of RPMI containing 10% FCS. After incubation for 24 h, non-migrating cells were removed from the upper surface of the membrane by scrubbing. Cells on the reverse side were stained with 0.1% crystal violet, and invading cells were counted under a microscope at 100× magnification [1,2].

**Western blot analysis**

Western blot analysis was performed as described previously [1,2]. Blots were incubated with the antibodies for SLC35B4 (NBP2-13329) and GAPDH (sc32233), followed by visualization using an enhanced chemiluminescence reagent (Amersham, Piscataway, NJ) and detected by VersaDoc 5000 (Bio-Rad Laboratories, Hercules, CA)

**References**

1. **Chiou JF, Tai CJ, Huang MT*, et al.*** Glucose-regulated protein 78 is a novel contributor to acquisition of resistance to sorafenib in hepatocellular carcinoma. *Ann Surg Oncol*. 2010; 17: 603-12.

2. **Chang YJ, Chiu CC, Wu CH*, et al.*** Glucose-regulated protein 78 (GRP78) silencing enhances cell migration but does not influence cell proliferation in hepatocellular carcinoma. *Ann Surg Oncol*. 2010; 17: 1703-9.

3. **Wang SK, Liang PH, Astronomo RD*, et al.*** Targeting the carbohydrates on HIV-1: Interaction of oligomannose dendrons with human monoclonal antibody 2G12 and DC-SIGN. *Proc Natl Acad Sci U S A*. 2008; 105: 3690-5.

4. **Sowinski S, Jolly C, Berninghausen O*, et al.*** Membrane nanotubes physically connect T cells over long distances presenting a novel route for HIV-1 transmission. *Nat Cell Biol*. 2008; 10: 211-9.

5. **Wei PL, Chang YJ, Ho YS*, et al.*** Tobacco-specific carcinogen enhances colon cancer cell migration through alpha7-nicotinic acetylcholine receptor. *Ann Surg*. 2009; 249: 978-85.

6. **Wei PL, Kuo LJ, Wang W*, et al.*** Silencing of glucose-regulated protein 78 (GRP78) enhances cell migration through the upregulation of vimentin in hepatocellular carcinoma cells. *Ann Surg Oncol*. 2012; 19 Suppl 3: S572-9.

**Table S1** Genotyped SNPs and the *P* values of their association with BCR after RP

| SNP ID | Chromosome | Position | BCR | | |
| --- | --- | --- | --- | --- | --- |
|  |  |  | Additive | Dominant | Recessive |
| rs505141 | 1 | 10488314 | 0.767 | 0.625 | 0.988 |
| rs6429759 | 1 | 15910003 | 0.601 | 0.774 | 0.526 |
| rs3768324 | 1 | 39492462 | 0.368 | 0.377 | 0.606 |
| rs785470 | 1 | 46519622 | 0.760 | 0.732 | 0.921 |
| rs3207053 | 1 | 53832824 | 0.198 | 0.486 | 0.104 |
| rs1264899 | 1 | 111992095 | 0.588 | 0.371 | 0.596 |
| rs10913672 | 1 | 178981416 | 0.266 | 0.402 | 0.302 |
| rs12071085 | 1 | 200123430 | 0.989 | 0.377 | 0.255 |
| rs10920236 | 1 | 201684221 | 0.508 | 0.740 | 0.401 |
| rs774139 | 1 | 220908088 | 0.559 | 0.566 | 0.742 |
| rs6727303 | 2 | 38025672 | 0.351 | 0.483 | - |
| rs4672016 | 2 | 55324960 | 0.178 | 0.467 | 0.050 |
| rs6754629 | 2 | 65142110 | 0.884 | 0.660 | 0.411 |
| rs13017338 | 2 | 86501553 | 0.215 | 0.084 | 0.818 |
| rs4399734 | 2 | 113072488 | 0.678 | 0.293 | 0.319 |
| rs6541775 | 2 | 122187367 | 0.625 | 0.326 | 0.611 |
| rs16832394 | 2 | 136743310 | 0.885 | 0.811 | 0.374 |
| rs4264585 | 2 | 191334031 | 0.880 | 0.928 | 0.873 |
| rs1020118 | 2 | 224896438 | 0.562 | 0.910 | 0.323 |
| rs2071203 | 3 | 50311900 | 0.775 | 0.833 | 0.330 |
| rs12487377 | 3 | 121361646 | 0.296 | 0.419 | 0.325 |
| rs4683404 | 3 | 141160371 | 0.501 | 0.487 | 0.775 |
| rs4686710 | 3 | 185633136 | 0.387 | 0.539 | 0.415 |
| rs1990676 | 3 | 186784638 | 0.544 | 0.312 | - |
| rs1678272 | 4 | 8353829 | 0.345 | 0.663 | - |
| rs3756205 | 4 | 25312615 | 0.956 | 0.933 | - |
| rs10938519 | 4 | 48038584 | 0.973 | 0.627 | 0.419 |
| **rs60701** | 5 | 10733776 | **0.025** | **0.001** | 0.910 |
| rs1421094 | 5 | 39355591 | 0.656 | 0.499 | - |
| rs12515069 | 5 | 81402224 | 0.132 | 0.135 | 0.480 |
| rs12109860 | 5 | 102393112 | 0.846 | 0.573 | 0.306 |
| rs1143684 | 6 | 3010390 | 0.309 | 0.724 | 0.103 |
| rs203877 | 6 | 28048624 | 0.567 | 0.514 | 0.838 |
| rs1570759 | 6 | 33633940 | 0.887 | 0.345 | 0.461 |
| rs12203953 | 6 | 36624422 | 0.453 | 0.546 | 0.531 |
| rs3749863 | 6 | 45345248 | 0.274 | 0.388 | 0.349 |
| **rs9395785** | 6 | 52217185 | 0.149 | **0.011** | 0.088 |
| **rs1883136** | 6 | 111907023 | **0.011** | **0.018** | 0.083 |
| rs4709360 | 6 | 157952160 | 0.344 | 0.679 | 0.231 |
| rs2286681 | 7 | 6035428 | 0.551 | 0.678 | 0.546 |
| rs6959646 | 7 | 29448660 | 0.404 | 0.120 | 0.531 |
| rs10953106 | 7 | 93671907 | 0.104 | 0.135 | 0.280 |
| rs11761361 | 7 | 102107697 | 0.371 | 0.804 | 0.191 |
| rs2242029 | 7 | 128695279 | 0.530 | 0.093 | 0.392 |
| **rs1646724** | 7 | 134001875 | **0.006** | **0.039** | **0.005** |
| rs2740444 | 8 | 11648789 | 0.135 | 0.059 | 0.901 |
| rs10955293 | 8 | 103809995 | 0.499 | 0.697 | 0.394 |
| rs10972047 | 9 | 34310357 | 0.722 | 0.469 | 0.881 |
| rs10972567 | 9 | 35728019 | 0.590 | 0.118 | 0.366 |
| rs12237222 | 9 | 85834743 | 0.257 | 0.290 | 0.508 |
| rs2795490 | 9 | 100909996 | 0.677 | 0.673 | 0.786 |
| rs2472473 | 9 | 107537681 | 0.841 | 0.934 | - |
| rs16911576 | 9 | 125026861 | 0.971 | 0.810 | 0.641 |
| rs359592 | 9 | 128047286 | 0.815 | 0.747 | 0.968 |
| rs3808836 | 9 | 130159059 | 0.675 | 0.508 | 0.940 |
| rs12634 | 9 | 132573536 | 0.654 | 0.889 | - |
| rs3124779 | 9 | 136281966 | 0.882 | 0.623 | 0.217 |
| rs1018330 | 9 | 139980385 | 0.469 | 0.248 | 0.886 |
| rs718641 | 10 | 11779908 | 0.288 | 0.278 | 0.535 |
| rs3740286 | 10 | 90751340 | 0.763 | 0.991 | 0.607 |
| rs807051 | 10 | 102802570 | 0.364 | 0.642 | 0.281 |
| rs7092340 | 10 | 104529668 | 0.853 | 0.537 | 0.452 |
| rs284857 | 10 | 104574063 | 0.348 | 0.992 | 0.090 |
| rs10835189 | 11 | 27541995 | 0.290 | 0.104 | 0.984 |
| rs4752979 | 11 | 47339180 | 0.531 | 0.952 | 0.209 |
| rs576483 | 11 | 59893606 | 0.704 | 0.632 | - |
| rs681309 | 11 | 65151449 | 0.208 | 0.182 | 0.679 |
| rs490998 | 11 | 66318972 | 0.303 | 0.461 | 0.331 |
| rs2253658 | 11 | 68856804 | 0.101 | 0.094 | 0.476 |
| rs921675 | 11 | 68869034 | 0.478 | 0.902 | 0.290 |
| rs3019751 | 11 | 68923597 | 0.475 | 0.800 | 0.304 |
| rs560354 | 11 | 126063192 | 0.703 | 0.985 | 0.461 |
| rs11048722 | 12 | 26999204 | 0.649 | 0.927 | 0.183 |
| rs3759148 | 12 | 49259958 | 0.264 | 0.445 | 0.289 |
| rs10876268 | 12 | 52676755 | 0.659 | 0.715 | 0.712 |
| **rs10859812** | 12 | 95374544 | 0.561 | **0.045** | 0.131 |
| rs4766664 | 12 | 113362997 | 0.833 | 0.869 | - |
| **rs2240317** | 12 | 120586193 | **0.007** | **0.013** | **0.048** |
| rs3916065 | 12 | 120934624 | 0.952 | 0.752 | 0.825 |
| rs9533339 | 13 | 43571021 | 0.720 | 0.997 | 0.474 |
| rs4942830 | 13 | 50019376 | 0.676 | 0.733 | 0.738 |
| rs1886543 | 13 | 52701782 | 0.959 | 0.986 | 0.902 |
| rs1950501 | 14 | 24806800 | 0.598 | 0.667 | 0.668 |
| rs1952013 | 14 | 50087357 | 0.487 | 0.579 | - |
| rs7146558 | 14 | 50784919 | 0.171 | 0.335 | 0.179 |
| rs12587742 | 14 | 73393391 | 0.505 | 0.734 | 0.395 |
| rs1076458 | 14 | 73393878 | 0.561 | 0.343 | 0.586 |
| rs1861423 | 14 | 77187061 | 0.228 | 0.521 | 0.188 |
| rs12897338 | 14 | 100844368 | 0.802 | 0.830 | 0.844 |
| rs11846404 | 14 | 104156108 | 0.452 | 0.367 | 0.776 |
| rs12440045 | 15 | 41782684 | 0.328 | 0.331 | - |
| rs1106934 | 15 | 42072623 | 0.787 | 0.938 | 0.651 |
| rs2301826 | 15 | 91525197 | 0.353 | 0.778 | 0.219 |
| rs7171542 | 15 | 101750459 | 0.698 | 0.646 | 0.948 |
| rs1053871 | 16 | 3181963 | 0.953 | 0.879 | - |
| rs4781062 | 16 | 11401416 | 0.714 | 0.833 | 0.649 |
| rs11075256 | 16 | 15187912 | 0.369 | 0.549 | 0.306 |
| rs4788084 | 16 | 28539848 | 0.062 | 0.147 | 0.088 |
| rs11859006 | 16 | 57422519 | 0.391 | 0.559 | 0.369 |
| rs12938667 | 17 | 1898800 | 0.221 | 0.239 | 0.493 |
| rs1662680 | 17 | 6952764 | 0.650 | 0.696 | 0.746 |
| rs9891938 | 17 | 15915072 | 0.961 | 0.614 | 0.335 |
| rs2257609 | 17 | 18865181 | 0.124 | 0.238 | 0.177 |
| **rs12948881** | 17 | 19637540 | **0.020** | **0.048** | 0.075 |
| rs1859407 | 17 | 20078137 | 0.802 | 0.651 | - |
| **rs9303642** | 17 | 29578360 | **0.023** | **0.002** | 0.593 |
| rs2227319 | 17 | 38170845 | 0.421 | 0.619 | 0.416 |
| rs4987082 | 17 | 47481374 | 0.385 | 0.123 | 0.602 |
| rs8071916 | 17 | 56660279 | 0.683 | 0.981 | 0.474 |
| rs2665831 | 17 | 61912392 | 0.620 | 0.695 | 0.674 |
| rs9902795 | 17 | 71188283 | 0.396 | 0.218 | 0.901 |
| rs11663049 | 18 | 12991393 | 0.101 | 0.125 | - |
| **rs317807** | 18 | 55250670 | 0.079 | 0.317 | **0.037** |
| rs4808485 | 19 | 16439498 | 0.307 | 0.654 | 0.217 |
| rs12983093 | 19 | 30199665 | 0.665 | 0.670 | 0.805 |
| rs2960321 | 19 | 39048163 | 0.993 | 0.984 | 0.985 |
| rs862458 | 19 | 39341182 | 0.811 | 0.778 | - |
| **rs4239504** | 19 | 40728036 | 0.838 | 0.279 | **0.009** |
| rs2288912 | 19 | 45449199 | 0.829 | 0.432 | 0.355 |
| rs2288478 | 19 | 49866919 | 0.612 | 0.886 | 0.473 |
| rs6509724 | 19 | 53596169 | 0.898 | 0.898 | - |
| rs36615 | 19 | 54705207 | 0.381 | 0.416 | - |
| rs6033860 | 20 | 1448575 | 0.732 | 0.410 | 0.335 |
| **rs2077109** | 20 | 37419286 | **0.011** | 0.091 | **0.010** |
| rs4629 | 20 | 44452697 | 0.967 | 0.819 | 0.871 |
| rs9982122 | 21 | 40547310 | 0.836 | 0.599 | 0.806 |
| rs2272576 | 21 | 40684389 | 0.617 | 0.262 | 0.609 |
| rs2839203 | 21 | 47716389 | 0.956 | 0.736 | 0.776 |
| rs737923 | 22 | 19132325 | 0.360 | 0.462 | 0.435 |
| rs5759603 | 22 | 23494833 | 0.231 | 0.462 | 0.185 |
| **rs5753355** | 22 | 31259658 | 0.393 | 0.631 | **0.031** |

Abbreviations: SNP, single nucleotide polymorphism; BCR, biochemical recurrence; RP, radical prostatectomy.

*P* values for log-rank test.

*P* < 0.05 is in boldface.
